# Axl regulated survival/proliferation network and its therapeutic intervention in mouse models of glomerulonephritis

**DOI:** 10.1186/s13075-022-02965-w

**Published:** 2022-12-28

**Authors:** Yuxuan Zhen, Yan Ren, Mario Medvedovic, David E. Adams, Diping Wang, Wen-Hai Shao

**Affiliations:** 1 Division of Immunology, Allergy and Rheumatology, Department of Internal Medicine, University of Cincinnati, 231 Albert Sabin Way, Cincinnati, OH 45267 USA; 2 Division of Biostatistics and Bioinformatics, Department of Environmental Health, University of Cincinnati, Cincinnati, OH 45267 USA; 3 Department of Pathology, University of Cincinnati, Cincinnati, OH 45267 USA

**Keywords:** Anti-GBM nephritis, Axl receptor tyrosine kinase, Lupus nephritis, R428, T cell infiltration, BUN

## Abstract

**Background:**

Lupus nephritis (LN) is the most common and serious complication of systemic lupus erythematosus (SLE). LN pathogenesis is not fully understood. Axl receptor tyrosine kinase is upregulated and contributes to the pathogenic progress in LN. We have reported that Axl disruption attenuates nephritis development in mice.

**Methods:**

In this study, we analyzed the gene expression profiles with RNA-seq using renal cortical samples from nephritic mice. Axl-KO mice were bred onto a B6.lpr spontaneous lupus background, and renal disease development was followed and compared to the Axl-sufficient B6.lpr mice. Finally, anti-glomerular basement membrane (anti-GBM) Ab-induced nephritic mice were treated with Axl small molecule inhibitor, R428, at different stages of nephritis development. Blood urine nitrogen levels and renal pathologies were evaluated.

**Results:**

Transcriptome analysis revealed that renal Axl activation contributed to cell proliferation, survival, and motility through regulation of the Akt, c-Jun, and actin pathways. Spontaneous lupus-prone B6.lpr mice with Axl deficiency showed significantly reduced kidney damages and decreased T cell infiltration compared to the renal damage and T cell infiltration in Axl-sufficient B6.lpr mice. The improved kidney function was independent of autoAb production. Moreover, R428 significantly reduced anti-GBM glomerulonephritis at different stages of GN development compared to the untreated nephritic control mice. R428 administration reduced inflammatory cytokine (IL-6) production, T cell infiltration, and nephritis disease activity.

**Conclusions:**

Results from this study emphasize the important role of Axl signaling in LN and highlight Axl as an attractive target in LN.

## Introduction

Lupus nephritis (LN) is a severe manifestation of systemic lupus erythematosus (SLE) that has significant comorbidity and mortality even with advanced therapies [1]. LN pathogenesis is complex and incompletely understood. Therapy is predominantly nonspecific immunosuppressive drugs that are only partly successful at suppressing disease and often cause severe adverse side effects [2, 3]. MRL/*lpr* spontaneous lupus mice have a recessive autosomal mutation of the Fas receptor and are characterized by lymphadenopathy due to an accumulation of CD3^+^CD4^−^CD8^−^ double-negative (DN) T cells [4]. These mice also developed elevated levels of autoAbs such as anti-dsDNA, resulting in large amounts of immune complexes [4]. Similarly, B6.lpr mice develop lymphoproliferation, pathological autoreactive immune responses, and lupus nephritis at a later stage [5]. C57BL/6 J.lpr (B6.lpr) strain is used widely as it shares the same genetic background as C57BL/6 with a large number of gene-manipulated mice [6].

Axl receptor tyrosine kinase is a member of the TAM family, along with Mer and Tyro-3. Growth arrest-specific protein 6 (Gas6) is the ligand that binds to Axl with high affinity [7]. Gas6 and Axl mRNA, but not protein, is detected in normal kidneys [8]. They are quickly upregulated upon kidney inflammation. Overexpression of Axl is also implicated in many cancer cells, including renal cell carcinoma (RCC) [9] and in tubular cells [10]. Gas6 and Axl expression and signaling actively participate in mouse and human renal diseases [11–15]. Serum Axl levels predict therapeutic responses and long-term outcomes in lupus nephritis [16]. Mesangial cell Axl activation led to Akt phosphorylation, which synergistically activates mTORC1 and NF-κB, resulting in cell proliferation and Bcl-xl upregulation, respectively [11]. Disruption of Gas6 expression or inhibition of Gas6 activity with warfarin led to less renal injury and improved survival in mice with nephrotoxic nephritis [17, 18]. In anti-glomerular basement membrane (anti-GBM)-induced nephritis, mice with Axl deficiency showed ameliorated kidney damage and decreased mesangial cell proliferation compared with the Axl-sufficient mice [15]. Most importantly, nephritic mice pre-treated with Axl small molecule inhibitor R428 (bemcentinib) had significantly improved kidney inflammation compared to untreated nephritic mice [14]. Here, we further investigated the protective role of Axl deficiency in the B6.lpr spontaneous lupus nephritis, which more closely resembles human lupus nephritis. We revealed major pathways attributed to Axl deficiency in nephritis and showed that targeting Axl may be clinically applicable, because mice treated with R428 after disease onset showed significantly improved kidney function and reduced pathological damages.

## Materials and methods

### Antibodies

Goat anti-mouse Axl antibody was purchased from R&D Biosystems, Inc. (Minneapolis, MN). All antibodies in Western blot were purchased from Cell Signaling Technologies (Boston, MA). Antibodies in flow cytometry analysis were purchased from BD Biosciences (Ann Arbor, MI). Axl small molecule inhibitor, R428 (bemcentinib), was from ApexBio (Boston, MA). Anti-GBM antisera (PTX-001S) were purchased from Probetex, Inc. (San Antonio, TX).

### Mice

B6.lpr/Axl-KO mice were generated by crossing B6.lpr mice (Jackson Laboratory, Bar Harbor, ME) with B6.Axl-KO mice (Dr. Carla Rothlin at Yale University). Genetic deficiency was confirmed by PCR-based genotyping and Western blotting. Mice were followed for up to 10 months of age with autoantibody titers and urinalysis for proteinuria. At the termination of the experiment, serum samples were collected, and spleens were removed and processed for FACS analysis. Kidneys were processed for formalin fixation or OCT embedding. Mice were housed under pathogen-free conditions at the animal facilities of the University of Cincinnati. Only female mice were included in the experiments. All animal experiments were performed in accordance with the guidelines of the Institutional Animal Care and Use Committee (IACUC).

### Induction of nephrotoxic nephritis with anti-GBM sera and R428 intervention

Mice (8-week-old female) were injected i.v. with 150 μl of anti-GBM sera on day − 2 and day 0. Control mice were given the same dose of normal sera. The severity of nephritis was followed by proteinuria dip sticks every 2–3 days and by measuring blood urine nitrogen (BUN, Urea Nitrogen Direct kit, Stanbio Laboratory, Boerne, TX). For the R428 treatment, mice were administered with 100 μl oral gavage of R428 (125 mg/kg, every other day), starting at day 3 (Fig. 3A) or day 8 (Fig. 4A). Blood samples were collected from the retro-orbital vein of the experimental mice. At termination (day 21), renal function was evaluated by histological analysis and immunofluorescent staining.

### Renal histology

Paraffin-embedded kidney sections (4 μm) were processed for hematoxylin and eosin (H&E), periodic-acid Schiff (PAS), and trichrome at the University of Cincinnati Pathology Core Center. Kidney histology was evaluated in a blind manner by a pathologist. The GN disease activity was assessed using the NIH-modified activity and chronicity indices, including global and/or segmental sclerosis in < 25% (1 +), 25–50% (2 +), or > 50% (3 +) of glomeruli; fibrous crescents in < 25% (1 +), 25–50% (2 +), or > 50% (3 +) of glomeruli; tubular atrophy in < 25% (1 +), 25–50% (2 +), or > 50% (3 +) of the cortical tubules; and interstitial fibrosis in < 25% (1 +), 25–50% (2 +), or > 50% (3 +) in the cortex [19].

### Immunofluorescent staining

OCT-embedded cryo kidney sections (4 μm) were fixed in acetone for 20 min and blocked with 10% BSA for 1 h. Sections were then incubated with rabbit anti-mouse C3 (Cat: PA1-40,288), APC conjugated anti-mouse CD4 (Cat: 17–0042-82), Alexa-488 conjugated anti-sheep IgG (Cat: A11015), or Alexa-488 conjugated anti-mouse IgG (Cat: A21202), for 1 h at room temperature, followed by Rhodamine-conjugated anti-rabbit (Cell Signaling Technology Beverly, MA. Cat: R6394, 1:100 dilution) for C3 secondary antibodies staining for 1 h. All primary Abs were purchased from Life Technologies (Carlsbad, CA, 1:100 dilution). Slides were covered with Fluoromount-G mounting medium (SouthernBiotech, Birmingham, AL) after three times washing with PBS. Images were acquired with a Leica DMi8 fluorescent microscope (Buffalo Grove, IL) and analyzed with the LAX S software (Leica Microsystems Inc.). CD4^+^ T cell numbers were counted in four (200 ×) low-power fields in each section. Sections from at least 4 mice were counted and analyzed.

### RT-PCR

Total RNA was isolated from the kidney cortex of experimental mice using the Invitrogen RNAqueous Total RNA Isolation Kit (Fisher Scientific, Waltham, MA). The first strand DNA was synthesized from 2 μg of total RNA using the High Capacity RNA-to-cDNA Kit (Applied Biosystems, Waltham, MA). Each cDNA sample was amplified using pre-made primers for mIL-6 (Mm00446190, Thermo Fisher Scientific, Waltham, MA). The housekeeping gene GAPDH (Mm99999915) was assayed as an internal control. Real-time PCR was performed using the StepOnePlus real-time PCR system with the TaqMan Gene Expression Master Mix (Applied Biosystems). The C_T_ of each test message was first normalized using the C_T_ for GAPDH, assayed in the same sample. Fold changes were then calculated using the relative C_T_ method: fold change = 2^(normalized C^_T_^from experimental mice − normalized C^_T_^from control mice)^.

### Anti-dsDNA autoantibody ELISA

Anti-dsDNA ELISA was performed as previously described [20]. Briefly, 96-well plates were pre-coated with poly l-lysine (1 μg/ml, Sigma-Aldrich, St. Louis, MO) for 1 h at room temperature. Double-stranded DNA (2.5 μg/ml, prepared from calf thymus) was added into each well after washing and incubated overnight at 4 °C. After washing and 2 h blocking, serum samples (1/250 diluted) were added and incubated overnight at 4 °C. AP-conjugated goat anti-mouse IgG (Jackson ImmunoResearch Lab) was added as the secondary Ab. The plates were washed and incubated with the PNPP substrate (Sigma-Aldrich, St. Louis, MO) for 30 min at 37 °C. The plates were read at various time points with the Epoch BioTek plate reader (Winooski, VT).

### Flow cytometry analysis of splenocytes

Spleen samples were isolated from the experimental mice. Single-cell suspensions were incubated with blocking buffer containing 2.4G2 for 45 min and followed by immunofluorescence-conjugated antibody incubation as specified in the figure legend. Samples were then washed twice with FACS buffer (1 × PBS with 1% BSA). Data were acquired with the BD LSRFortessa cell analyzer (BD Biosciences) and analyzed with the FlowJo software (version 10.7.1, Ashland, OR).

### RNA-seq analysis of kidney cortex samples

Nephritis was induced in the B6.WT and B6.Axl-KO female mice (8 weeks old) as described in the “Materials and methods” section. Nephritis was confirmed with BUN and proteinuria. Mice were euthanized at day 8 after the second anti-GBM injection. Renal cortex samples from 2 B6.WT control, 2 B6.WT nephritic, and 3 B6.Axl-KO nephritic mice were analyzed, and there was no pooling of samples. Directional RNA-seq was performed at the Genomics, Epigenomics, and Sequencing Core (GES Core) of the University of Cincinnati. The RNA quality was measured by Bioanalyzer (Agilent, Santa Clara, CA). To isolate polyA RNA, NEBNext Poly(A) mRNA Magnetic Isolation Module (New England BioLabs, Ipswich, MA) was used with a total of 1 µg of good quality total RNA as input. The polyA RNA was enriched using SMARTer Apollo NGS library prep system (Takara Bio USA, Mountain View, CA). Next, NEBNext Ultra II Directional RNA Library Prep Kit (New England BioLabs) was used for library preparation, which is dUTP-based stranded library. The library was indexed and amplified under PCR cycle number of 8. After library Bioanalyzer QC analysis and quantification, individually indexed libraries were proportionally pooled and sequenced using Nextseq 550 sequencer (Illumina, San Diego, CA). Under the sequencing setting of single read 1 × 85 bp, about 25 million pass filter reads per sample were generated.

Sequence reads were aligned to the current reference mouse genome (GRCm37) using the STAR aligner [21], and the reads aligned to each known gene were counted based on the latest GENCODE definitions of gene features [22]. The quality control of raw and aligned reads was performed using FastQC [23], RNA-SeQC [24], and summarized using the MultQC [25] software. Differentially expressed genes were identified based on the FDR-adjusted *p*-values [26] obtained by fitting a generalized linear model based on the negative binomial distribution of read counts as implemented in the *edgeR* Bioconductor package [27]. The patterns of gene expression across different sample groups were summarized and visualized using Bayesian infinite mixture models [28–31] cluster analysis methods.

The functional characteristics of DEGs were studied by gene set enrichment analysis (GSEA) [32] as implemented in the R package *fgsea* of the Kyoto Encyclopedia of Genes and Genomes (KEGG) pathways [33, 34]. The mapping and visualization of the differences in the gene expression levels in the context of KEGG pathway networks were performed using the *pathview* Bioconductor package [35].

### Statistical analysis

Statistical analyses were performed using the unpaired Student’s *t* test with Welch’s correction. Data are presented as mean ± SEM. Quantitative figures and statistical analysis were performed using Prism 9 (graphPad). *p* < 0.05 was defined as statistically significant.

## Results

### Ameliorated nephritis in B6.lpr/Axl-KO mice is due to the renal intrinsic Axl function

**Fig. 1:**
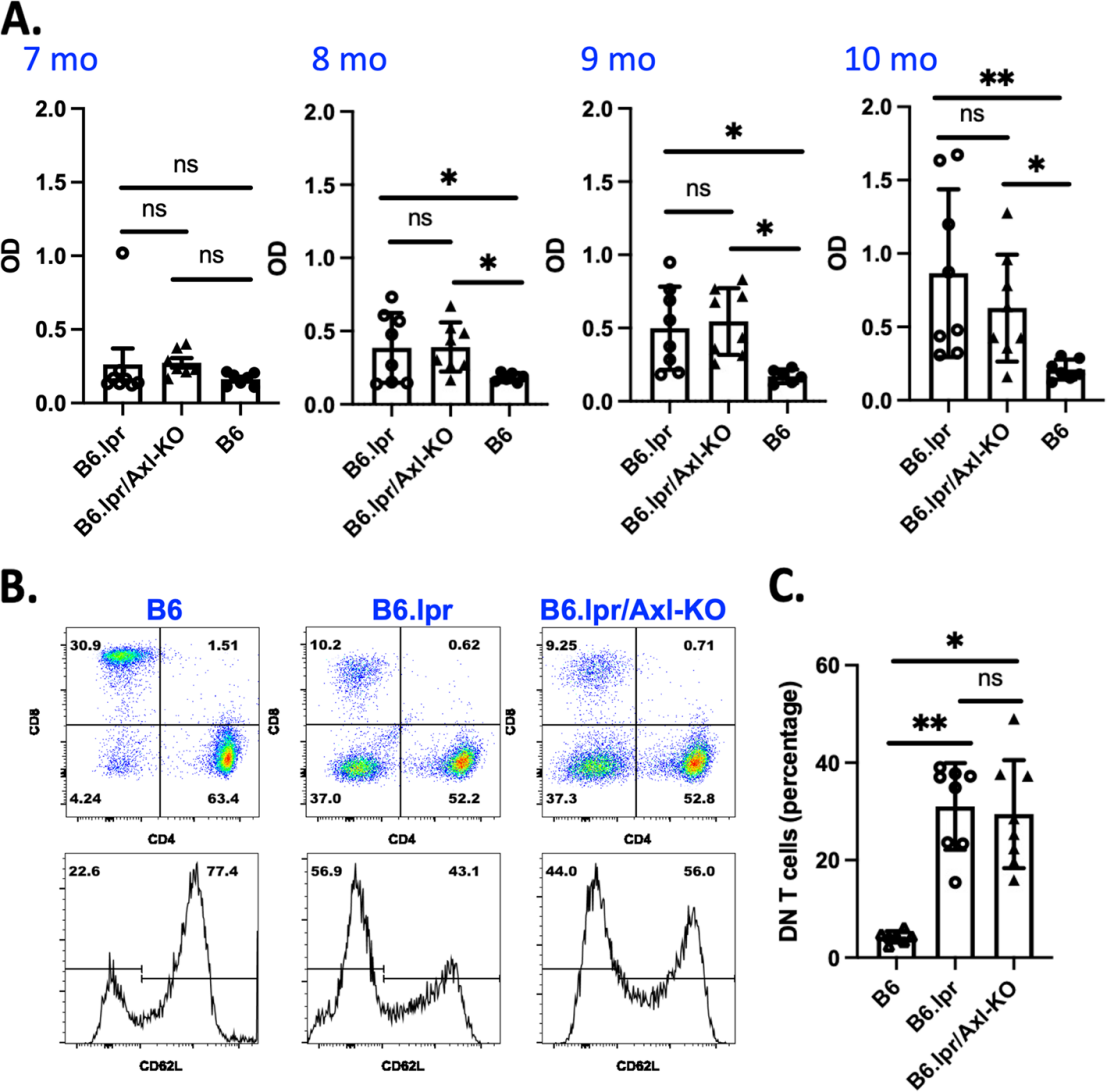
Similar systemic autoimmune responses in B6.lpr and B6.lpr/Axl-KO mice. Mice were generated as described in the “Materials and methods” section. **A** Blood samples (*n* = 8 for each group) were collected at the age of 7, 8, 9, and 10 months. Serum anti-dsDNA levels were measured by ELISA. Antibody levels were presented as OD. **B** The spleens were removed from experimental animals. Single-cell splenocytes were prepared in FACS buffer and processed for surface staining. Data were acquired with the Forsseta LSR-II flow cytometer and quantified in **C**. Two sets of experimental mice (a total of 16 mice) were followed. Similar results were observed. Data presented are from one set. Statistical analysis was performed with an unpaired *t* test with Welch’s correction. ns, not significant; *p < 0.05; **p < 0.01

We previously showed improved renal inflammation and proteinuria in anti-GBM-induced glomerulonephritis (GN) in B6.Axl-KO mice [15]. Therapeutically targeting the Axl/Akt pathway improved renal function in the same model [14]. However, the spontaneous B6.lpr lupus mice develop a full spectrum of lupus disease manifestation, closely resembling human SLE [6]. We wondered if the same protection in GN by Axl deficiency emerges in spontaneous lupus mice. Nephritis was followed for up to 10 months. At this age, B6.lpr mice developed high titer anti-dsDNA autoAbs and accumulated the DN T cells in the lymphoid organs [36] (Fig. 1). However, the anti-dsDNA autoAb levels were found to be not significantly different in the B6.lpr and B6.lpr/Axl-KO mice over the 4-month range followed (7, 8, 9, and 10 months) (Fig. 1A). DN T cells from spleen samples of B6.lpr and B6.lpr/Axl-KO mice were about 9 times higher than the DN T cells from the age-sex matched B6 WT control mice, but no statistic differences in DN T cell numbers between B6.lpr/Axl-KO and B6.lpr mice were observed (Fig. 1B upper row and 1C). CD4^+^ T cells were similarly activated in B6.lpr and B6.lpr/Axl-KO mice as shown by CD62L downregulation (Fig. 1B bottom row).

**Fig. 2:**
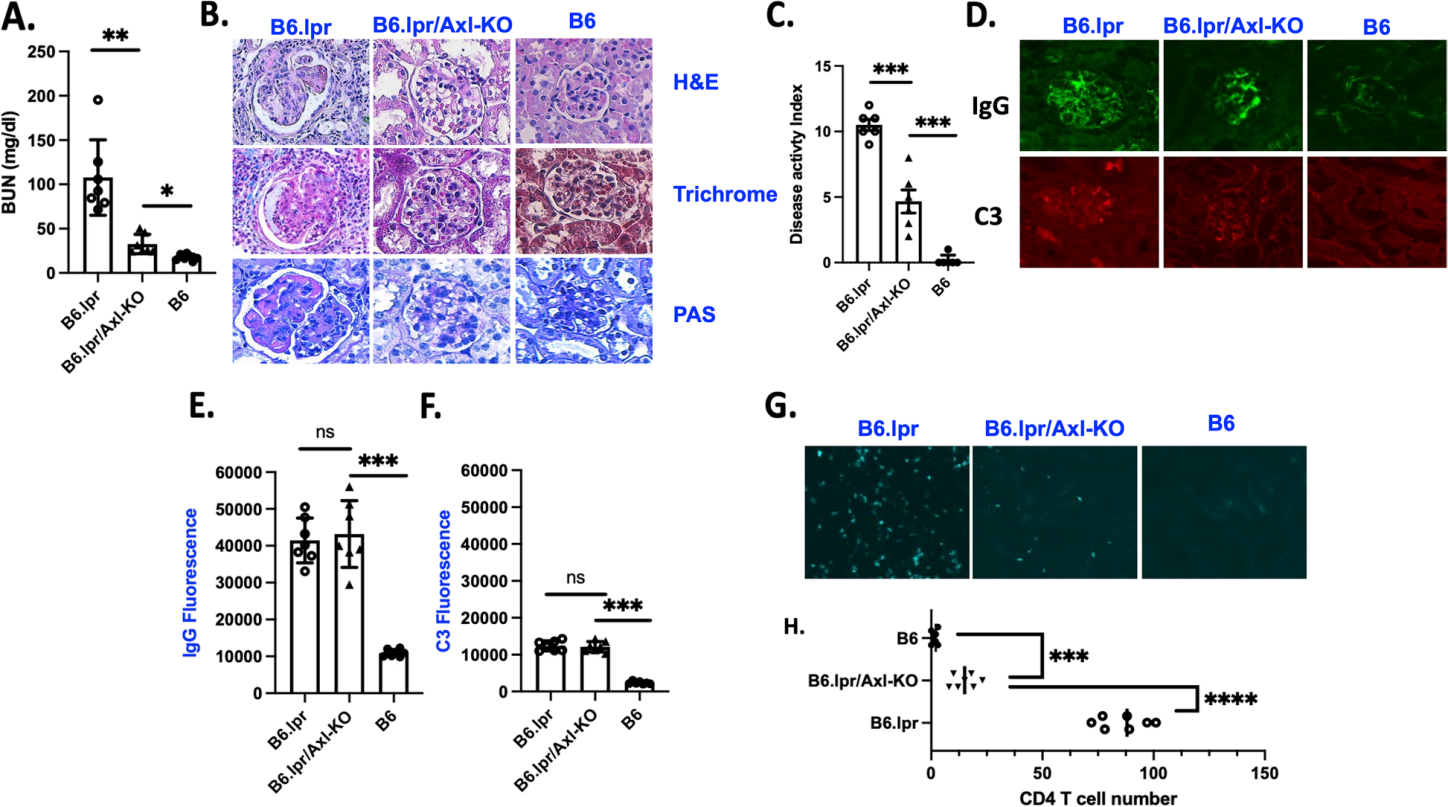
Ameliorated lupus nephritis in B6.lpr/Axl-KO mice. B6.WT (*n* = 7), B6.lpr (*n* = 7), and B6.lpr/Axl-KO (*n* = 7) mice at age of 10 months were euthanized. Blood samples were collected and processed for BUN (**A**) measurement. Kidney sections (4 μm) were processed for H&E, trichrome, and PAS staining (**B**). Disease activity was quantified in **C**. Immune complex and complement C3 (**D**) staining were performed and quantified (**E**, **F**). **G** CD4^+^ T cells were stained, and images were taken. T cell numbers per microscopic field were counted, and averaged numbers from each mouse were presented in **H**. Statistical analysis was performed with an unpaired *t* test with Welch’s correction. ns, not significant; **p < 0.01; ***p < 0.001. Representative images were shown at × 200 magnification

Further evaluation of kidney functions in 10-month-old mice revealed that B6.lpr mice developed severe nephritis, with a decline in kidney function indicated by increased BUN, immune complex deposition, and C3 deposition in B6.lpr mice [36] and (Fig. 2A–F). The glomeruli from B6.lpr mice displayed occluded and distended capillaries filled with PAS-positive fibrinous material. Proteinaceous tubular casts were evident. Kidney sections from B6.lpr mice were evident with enlarged glomerular Bowman’s space (H&E staining), segmental sclerosis (trichrome staining), and GBM thickening and fibrinoid necrosis (PAS staining) (Fig. 2B). However, B6.lpr/Axl-KO showed significantly decreased BUN levels (Fig. 2A) and milder renal damage (Fig. 2B, C), confirming the protective role of Axl deficiency in spontaneous lupus nephritis in mice.

No statistically significant differences in autoAb levels were observed between the two groups at all time points measured (Fig. 1A). Since immune complex deposition initiates and plays an important pathological role in LN of human SLE and mouse models of lupus [37–39], we wondered if the improved kidney function in B6.lpr/Axl-KO mice is due to a decreased immune complex deposition and associated complement activation. As shown in Fig. 2D, E, the overall immune complex measured by mouse IgG immunofluorescent staining showed no significant differences between B6.lpr and B6.lpr/Axl-KO mice at the age of 10 months. Similar levels of C3 deposition were also detected in the kidney sections of B6.lpr and B6.lpr/Axl-KO mice (Fig. 2D, F). These results suggest that improved kidney function in B6.lpr/Axl-KO mice is likely due to renal intrinsic Axl deactivation. This conclusion is also supported by studies that showed that Axl activation led to mesangial cell proliferation and survival during GN [11, 18, 40].

T cells infiltrate the kidney to injure renal cells directly via cytotoxicity or indirectly through activation and recruitment of other cells [41]. Immunofluorescent staining of the kidney sections from 10-month-old experimental mice showed a significant reduction of infiltrated CD4^+^ T cells in the kidney of B6.lpr/Axl-KO mice compared to T cell numbers in the kidney of B6.lpr mice (Fig. 2G, H).

### R428 treatment attenuates renal inflammation in GN

We recently showed in the anti-GBM GN that R428 prevents nephritis development [14]. This study tested whether Axl inhibition with R428 after nephritis onset can ameliorate renal inflammation, therefore improve kidney function. There are two phases of anti-GBM nephritis. The heterologous phase (days 2–5) is mediated by injected exogenous anti-GBM Abs and is complement-dependent; the autologous phase (day 6 and after) is mediated by endogenous Ab responses and is FcγR-dependent [42–44]. To identify the therapeutic window required for Axl inhibition to attenuate nephritis development in those mice, we administered R428 at day 3 and day 8 post-anti-GBM injection.

**Fig. 3:**
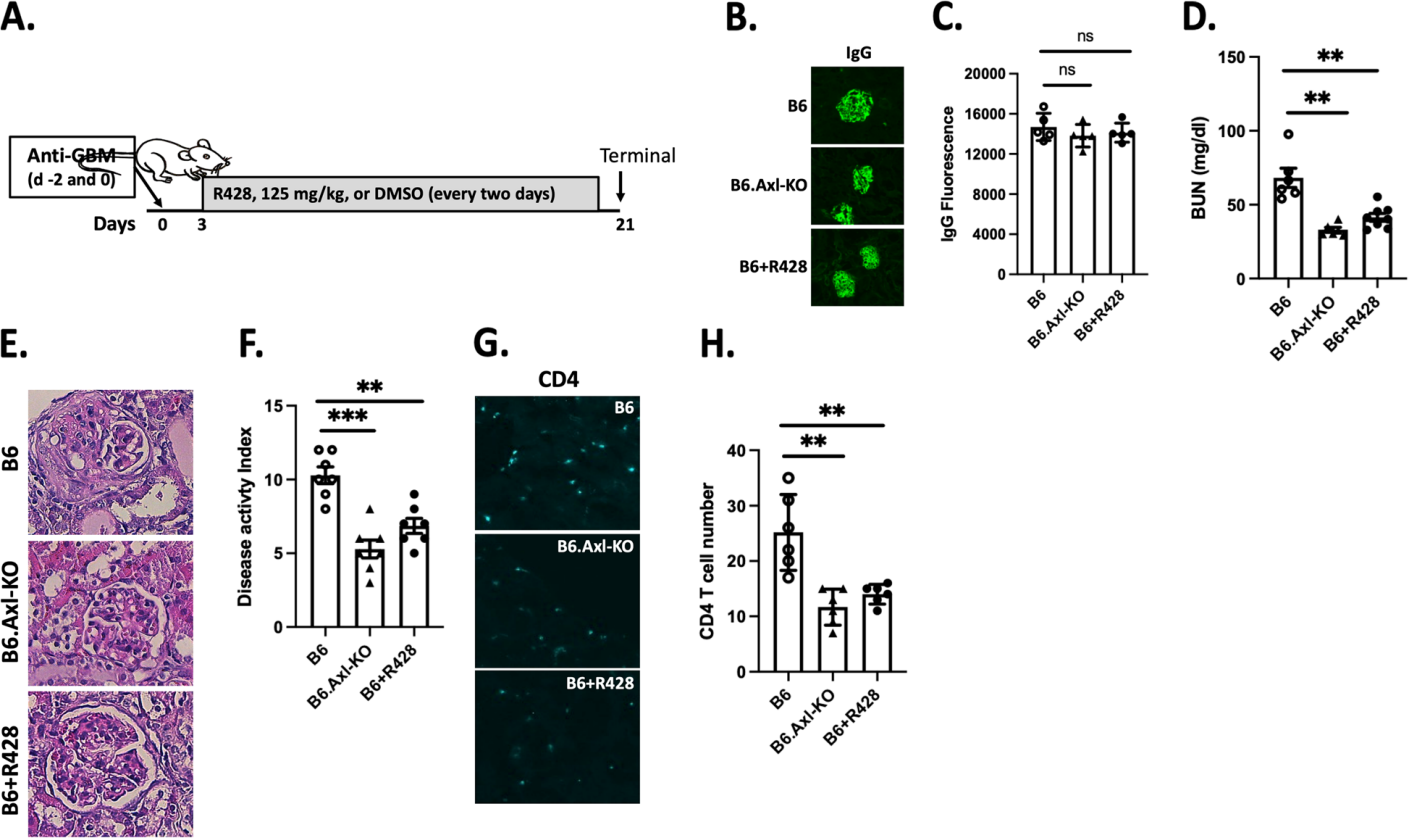
Ameliorated nephritis in mice with Axl-targeted early intervention. Three groups of mice (B6 (*n* = 6), B6.Axl-KO (*n* = 6), and B6 + R428 (*n* = 7)) were included. Nephritis was induced in experimental mice. The experiment was terminated on day 21. Blood, urine, and kidney samples were collected and processed for further analysis. **A** Schematic depiction of the treatment option with start time, dose, and interval. **B** Representative images for anti-sheep IgG immunofluorescent staining. **C** Quantitative analysis of the immunofluorescent intensity. **D** BUN levels were analyzed as depicted in the “Materials and methods” section. Kidney sections were processed for histology analysis (**E**, **F**). **G** CD4^+^ T cells were stained, and images were taken. T cell numbers per microscopic field were counted, and averaged numbers from each mouse were presented in **H**. Experiments were repeated three times. Similar data were obtained, and one set was presented here. Statistical analysis was performed with an unpaired *t* test with Welch’s correction. ns, not significant; **p < 0.01; ***p < 0.001. Representative images were shown at × 200 magnification

Three groups of female mice (8 weeks of age) were given anti-GBM at days − 2 and 0, and one group was treated with 125 mg/kg R428 starting at day 3 every other day (Fig. 3A). The B6 nephritic group treated with vehicle and the B6.Axl-KO nephritic group served as control groups. Treatment was terminated at day 21. We first measured sheep IgG deposition along the glomerular basement. No significant differences were detected, indicating equivalent amounts of anti-GBM sera were administered (Fig. 3B, C). As expected, vehicle-treated B6 control mice showed elevated BUN; in contrast, B6.Axl-KO mice showed significantly decreased BUN (Fig. 3D) as previously demonstrated [14, 15]. BUN levels from the R428-treated B6 mice were also significantly lower than that in vehicle-treated B6 mice (Fig. 3D). Kidney histology confirmed renal damages (Fig. 3E) in vehicle-treated nephritic B6 mice. Such histological changes were significantly reduced in B6.Axl-KO and R428-treated B6 mice (Fig. 3E). Overall renal disease activities were summarized in the disease activity index in Fig. 3F. Significantly reduced CD4^+^ T cell numbers were detected in the kidney of nephritic B6.Axl-KO mice compared to the T cell numbers in the kidney of nephritic B6 mice (Fig. 3G, H). R428-treated nephritic B6 mice also displayed a significantly reduced number of CD4^+^ T cells in the kidney compared to the T cell numbers in the kidney of nephritic B6 mice (Fig. 3G, H). These data demonstrate that R428 treatment can be effective at the heterologous phase of anti-GBM GN.

**Fig. 4:**
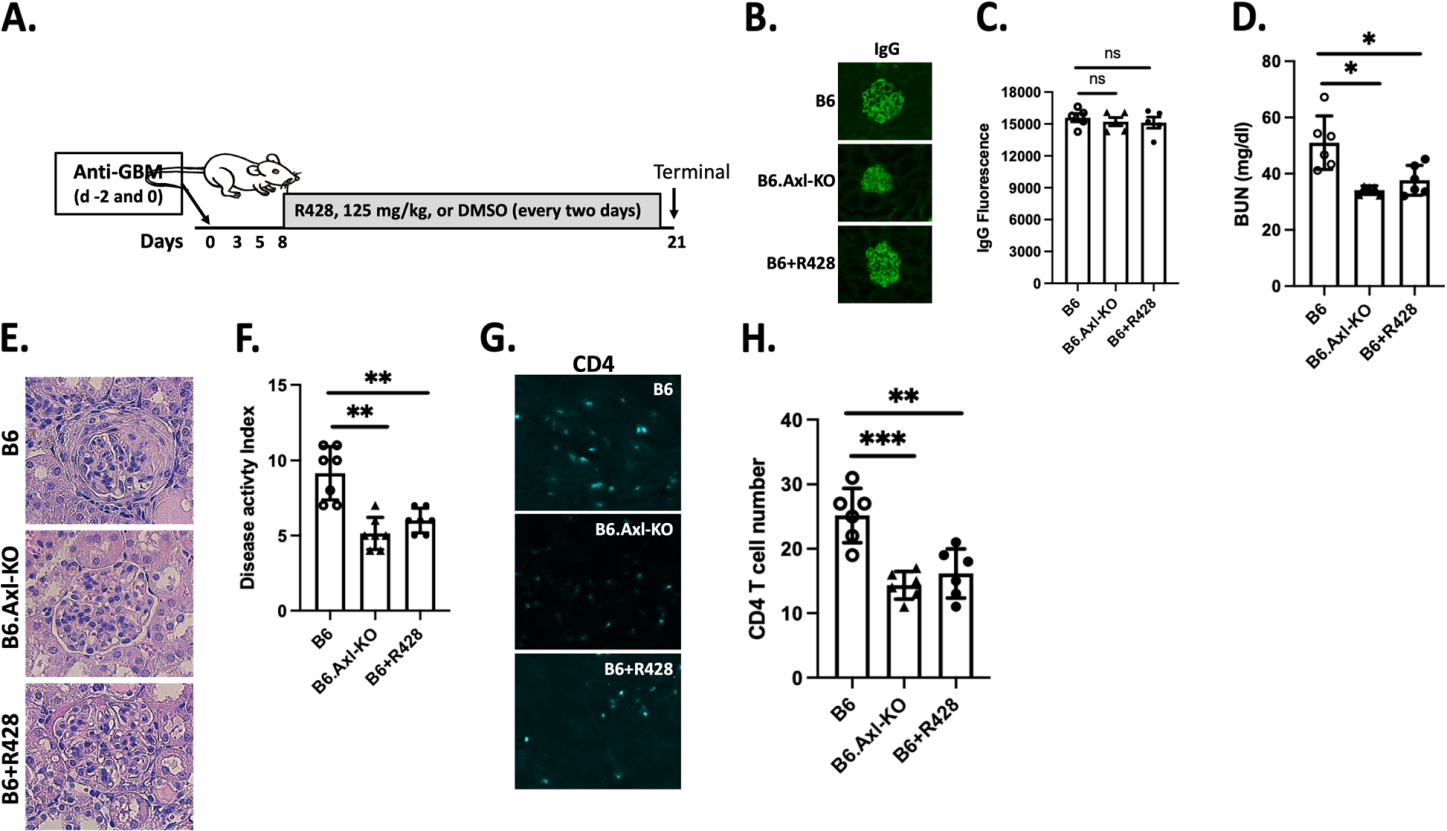
Improved nephritis in mice with Axl-targeted intervention started on day 8. Three groups of mice (B6 (*n* = 6), B6.Axl-KO (*n* = 6), and B6 + R428 (*n* = 6)) were included. Nephritis was induced in experimental mice. The experiment was terminated on day 21. Blood, urine, and kidney samples were collected and processed for further analysis. **A** Schematic depiction of the treatment option with start time, dose, and interval. **B** Representative images for anti-sheep IgG immunofluorescent staining. **C** Quantitative analysis of the immunofluorescent intensity. **D** BUN levels were analyzed as depicted in the “Materials and methods” section. Kidney sections were processed for histology analysis (**E**, **F**). **G** CD4^+^ T cells were stained, and images were taken. T cell numbers per view were counted and presented in **H**. Experiments were repeated two times. Similar data were obtained, and one set was presented here. Statistical analysis was performed with an unpaired *t* test with Welch’s correction. ns, not significant; *p < 0.05; **p < 0.01; ***p < 0.001. Representative images were shown at × 200 magnification

Next, we performed a similar experiment but treated the R428 group at day 8 post-nephritis induction (Fig. 4A). Similar amount of anti-GBM injection was first confirmed across the experimental groups by showing a similar intensity of sheep IgG deposition (Fig. 4B, C). As demonstrated in the previous experiment (Fig. 3), B6 mice developed GN disease and kidney pathology (Fig. 4D–F). B6.Axl-KO mice developed significantly reduced BUN and kidney injury (Fig. 4D–F). Most importantly, R428 treatment at the autologous phase (day 8) attenuated kidney inflammation as demonstrated by significantly decreased BUN (Fig. 4D), together with overall nephritis disease activity (Fig. 4E, F). CD4^+^ T cell numbers were also significantly reduced in the kidneys of B6.Axl-KO mice and R428-treated B6 mice compared to the T cell numbers in the kidneys of nephritic B6 mice (Fig. 4G, H). These results indicate that Axl small molecule inhibitor, R428, is efficient in attenuating nephritis development at both heterologous and autologous phases in the anti-GBM GN (Figs. 3 and 4).

### Axl inhibition reduces IL-6 production

**Fig. 5:**
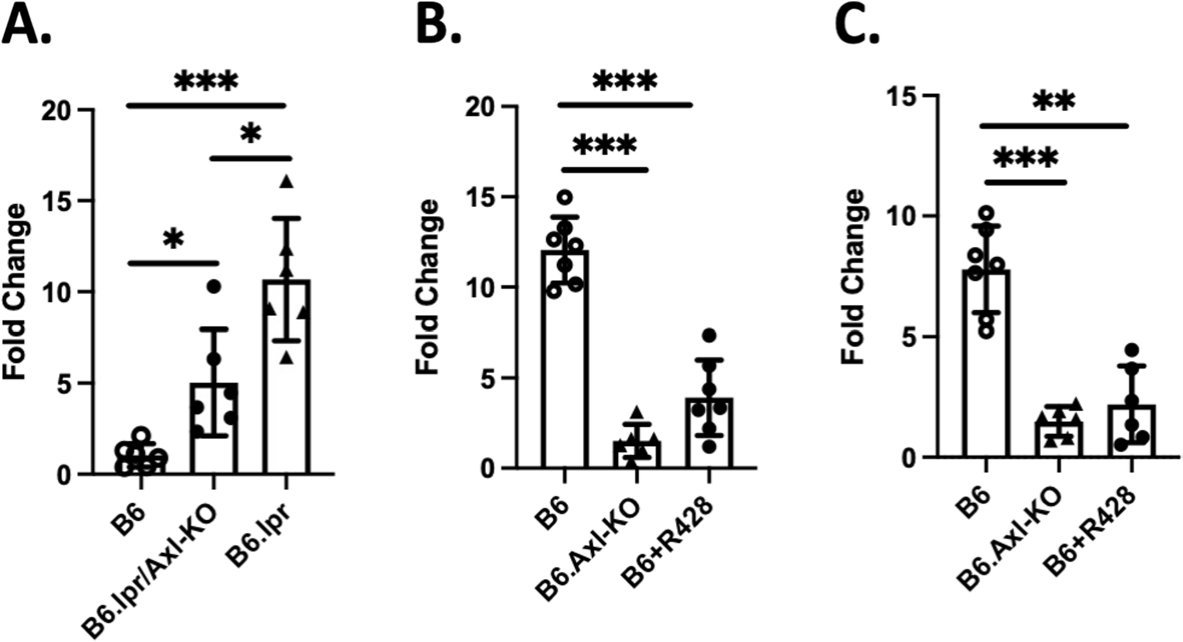
Reduced IL-6 levels in B6.lpr/Axl-KO mice and R428-treated nephritic B6 mice. Nephritis was induced in B6 and B6.Axl-KO mice with anti-GBM sera as previously described [47]. RNA samples were prepared from the renal cortex of experimental and B6 control mice and processed for RNA-seq analysis as described in the “Materials and methods” section. RT-PCR analysis of IL-6 expression in the cortex samples from experimental mice (as shown in Figs. 2, 3, and 4): either B6.lpr lupus mice (**A**) or mice treated with R428 at day 3 (**B**) and day 8 (**C**). Fold changes were then calculated using the relative C_T_ method: fold change = 2^(normalized C^_T_^from experimental mice − normalized C^_T_.^from control mice)^. Statistical analysis was performed with an unpaired *t* test with Welch’s correction. ns, not significant; *p < 0.05; **p < 0.01; ***p < 0.001

IL-6 plays a pathological role in GN and other forms of nephritis [45, 46]. We detected significantly decreased IL-6 levels in the kidney of nephritic B6.Axl-KO mice compared to the nephritic B6 Axl sufficient mice [15]. To investigate whether Axl deficiency or R428-treatment attenuated renal inflammation is associated with decreased IL-6 levels in the experimental mice, we performed IL-6 RT-PCR with kidney cortex mRNA extractions. We first measured IL-6 mRNA levels in the kidney samples from the 10-month B6.lpr and B6.lpr/Axl-KO mice. As shown in Fig. 5A, IL-6 mRNA levels in the kidney of 10-month B6.lpr/Axl-KO mice were significantly lower than that in the age/sex-matched kidney of B6.lpr WT mice. As expected, IL-6 levels in the kidneys of B6.lpr and B6.lpr/Axl-KO mice were significantly higher than that in the kidneys of age-matched non-diseased control B6 mice (Fig. 5A). There were also significantly decreased IL-6 mRNA levels in both day 3 and day 8 R428-treated groups compared to the vehicle-treated B6 groups, respectively (Fig. 5B, C). IL-6 mRNA levels were significantly lower in B6.Axl-KO nephritic mice compared to the IL-6 levels in the kidney of nephritic B6 mice (Fig. 5B, C). Data demonstrated attenuated inflammatory responses in the kidneys of both spontaneous and inducible nephritis mice when Axl function is disrupted by genetic manipulation or by a small molecule inhibitor, R428.

### Axl is involved in the survival, proliferation, and motility pathways in GN

**Fig. 6:**
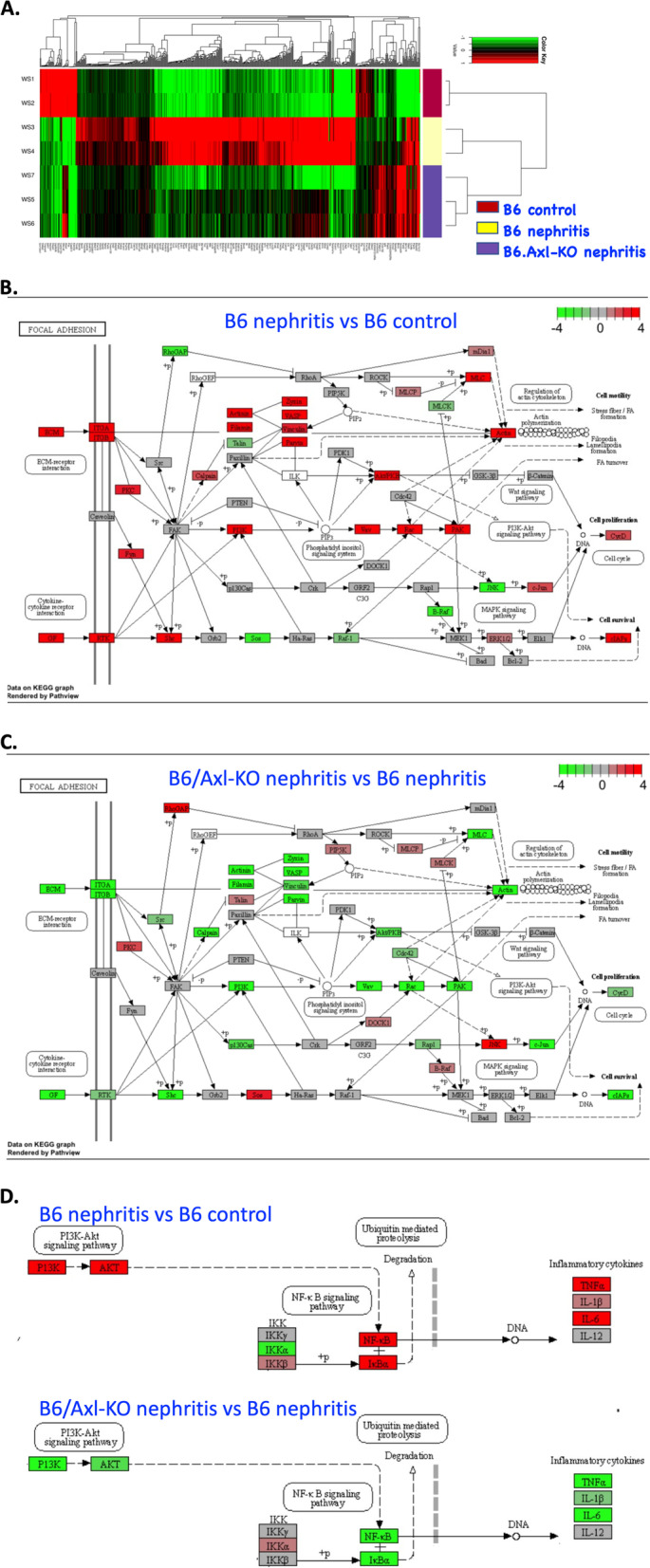
Molecular pathways regulated by Axl in glomerular nephritis. **A** Heat map of the expression patterns of responsive genes. Expression levels were centered by subtracting the average expression levels of a gene across all samples. Key molecular pathways altered in B6 nephritic kidney compared to B6 normal controls (**B**) and in B6.Axl-KO nephritic kidney compared to B6 nephritic kidney (**C**). **D**. PI3K pathway regulated IL-6 expression in nephritic mice from RNA-seq analysis. Values used in coloring pathway nodes were -log10 (fdr levels of the different expression), multiple by − 1 if the expression ratio was less than 1

To examine the molecular mechanism by which Axl activity regulates GN, we profiled a total of 15,715 transcriptomes of the cortex regions from B6.WT and B6.Axl-KO mouse kidneys at day 8 after anti-GBM nephritis. Compared to the cortex samples from the non-diseased B6 control mice, there were 437 significantly upregulated and 76 significantly downregulated genes in the cortex RNA preparations from WT nephritic mice (Fig. 6A, with a sixfold change and fdr < 0.1 cutoff). Compared to the nephritic cortex samples from the B6 nephritic mice, there were 147 significantly downregulated and 28 upregulated genes in the cortex samples from nephritic B6.Axl-KO mice (Fig. 6A, with a sixfold change and fdr < 0.1 cutoff). A gene expression profile in the nephritic B6.Axl-KO mice mostly resembled the pattern in B6.WT control mice for the significantly regulated genes (Fig. 6A).

To identify the most differentially regulated pathways, we performed the Kyoto Encyclopedia of Genes and Genomes (KEGG) pathway analysis. The final differences in the gene expression levels in the context of KEGG pathway networks were mapped and visualized using the *pathview* Bioconductor package. The results suggest that Axl might regulate three pathways (Akt, c-Jun, and actin) involving cell survival, proliferation, and motility (Fig. 6B, C). PI3 kinase (PI3K) seemed to be the major down-stream signaling stimulated by Axl activation (Fig. 6B, C), which has been demonstrated by studies with in vitro cell cultures and in animal models of GN and cancer [11, 48–51]. Focal adhesion kinase (FAK) has a central role in the pathway analysis, but FAK seemed not to be transcriptionally regulated by the Axl pathway as no change in the expression levels detected between Axl deficient and sufficient nephritic mice kidneys (compared Fig. 6B, C). Interestingly, c-Jun forms a complex with other TFs to drive Axl expression in cancer cells [52]. We also identified binding sites for transcription factors AP1 and c-Jun at the promoter region of the *Axl* gene [14]. Here, we demonstrated that c-Jun was upregulated in the kidneys of B6.WT nephritic mice at day 8 (Fig. 6B), at which time, Axl is also upregulated [15].

Consistent with a role for IL-6, RNA-seq data showed decreased IL-6 levels in the kidney of nephritic B6.Axl-KO mice compared to the nephritic B6.Axl-sufficient mice (Fig. 6D). Gene expression analysis indicated that IL-6 expression in the nephritic kidney is under the PI3K/Akt pathway and coactivated by the IKK degradation pathway (Fig. 6D).

## Discussion

A critical role of Axl signaling in kidney inflammation is well-known. Supporting the importance of Axl and suggesting a strategy for ameliorating LN, we now demonstrated for the first time that a small molecular inhibitor of Axl attenuates the onset of GN. The RNA transcriptome data not only confirmed the previously discovered cell survival and proliferation pathways regulated by Axl, but also revealed the potential for an effect on cell motility. The effectiveness of Axl inhibition in attenuating nephritis development at both phases highlights the vital role of Axl in GN and also suggests a broad therapeutic window for Axl-targeted treatment. Anti-GBM model is a powerful model of GN, providing insightful molecular mechanisms in nephritis development. However, spontaneous mouse lupus models more closely resemble human lupus and display the full spectrum of disease manifestation. Showing the efficacy of Axl inhibition in lupus-prone mouse models would be more relevant in guiding clinical application. Data from the B6.lpr/Axl-KO mice (Fig. 2) warrant further investigation.

T cells make up the majority of kidney infiltrating immune cells in lupus-prone mice [53] and play central and multiple roles in the pathogenesis of lupus nephritis [41, 54]. Reduced CD4^+^ T cells in the kidney of lupus-prone B6.lpr/Axl-KO mice and R428-treated anti-GBM nephritic mice suggest that they might partially contribute to the attenuated nephritis in those mice. Kidney infiltrating T cells appeared to be predominant in the peri-glomerular and interstitial areas and associated with kidney injury in patients with anti-GBM disease [55]. Similarly, T cells in the kidney of B6 nephritic mice also appeared to be in the peri-glomerular and interstitial areas (data not shown). However, Tilstra et al. reported that kidney-infiltrating T cells in spontaneous murine lupus nephritis mice are metabolically and functionally exhausted [53]. Those exhausted infiltrating T cells may retain some effector function. In addition, effector T cells co-exist with the exhausted T cells in the kidney of lupus nephritis mice [53]. Further studies are needed to characterize the effector or exhausted phenotype of these infiltrated CD4 T cells.

A striking observation is the marked effect of Axl inhibitor on IL-6 expression and pathways known to be affected by IL-6 signaling. IL-6 is a pleiotropic cytokine that regulates the inflammatory response in renal resident cells, including mesangial cells and tubular epithelial cells [46]. IL-6 and Axl expression are also regulated by a common transcription factor, AP1 [52, 56].  IL-6 and Axl have complex interactions with each other suggesting that they may have synergistic roles in renal inflammation. IL-6 binds to the non-signaling membrane-bound IL-6 receptor (IL-6R), which then activate gp130 to transduce IL-6 signaling. Mesangial cells do not express IL-6R but can be activated by the IL-6 and soluble IL-6R (sIL-6R) complex triggered gp130 activation. Interestingly, sIL-6R is cleaved from the membrane by the metalloproteases ADAM10 and 17, both of which are also responsible for Axl cleavage from the cell membrane [57]. Increased levels of soluble Axl (sAxl) have been associated with the severity of LN [58]. IL-6 activated STAT3 inhibits microRNA-134a [59], which is one of the mechanisms responsible for Axl regulation [60].

New development in clinical trials has led to FDA’s recent fast-track designation to bemcentinib (R428) in combination with the anti-PD-1/L1 agent for patients with non-small cell lung cancer. Encouraging data from the current study may facilitate the quick translation of bemcentinib treatment in LN patients, either alone or in combination with current standard therapies. However, more studies are needed to further optimize the benefits of Axl inhibition. The duration of Axl inhibition in ameliorated nephritis also needs to be evaluated.

## Conclusions

We showed the therapeutics of Axl inhibition in anti-GBM GN. We discovered major pathways attributed to Axl deficiency in GN mice. Nephritic mice treated with R428 exhibited attenuated renal inflammation and disease activity. Axl inhibition is beneficial at pre-disease [14] and post-disease phases in GN (Figs. 4 and 5). Additional mechanisms must be explored to minimize the necessary period of Axl inhibition and reduce renal injury from long-term treatment in the current clinical settings.

## Data Availability

All data generated or analyzed during this study are included in this published article.

## Abbreviations

DN: Double-negative
Gas6: Growth arrest-specific protein 6
GBM: Glomerular basement membrane
GN: Glomerulonephritis
H&E: Hematoxylin and eosin
LN: Lupus nephritis
PAS: Periodic-acid Schiff
SLE: Systemic lupus erythematosus
TAM: Tyro-3, Axl, and Mer receptor tyrosine kinases
